# PP52 Advancing Global Health Technology Assessment Practices: Evaluating Immediate Versus Five-Year Risk-Guided Cardiovascular Prevention Strategies In Australia

**DOI:** 10.1017/S0266462325102717

**Published:** 2025-12-29

**Authors:** Zanfina Ademi, Jedidiah Morton

## Abstract

**Introduction:**

Current Australian and global cardiovascular disease (CVD) prevention guidelines utilize a five-year risk assessment, potentially overlooking individuals with low short-term but high long-term CVD risk. This study evaluated the cost effectiveness of initiating intervention for risk factor control at age 40 years, regardless of calculated risk, compared with the conventional strategy recommended by guidelines, thereby advancing HTA to meet broader global health demands.

**Methods:**

We employed a novel causal-microsimulation model containing 108 varied risk factor profiles from the UK Biobank, each replicated 10,000 times, to project outcomes from age 40 to 85 years. The model evaluated the effects of early low-density lipoprotein cholesterol and blood pressure management versus beginning treatment at the conventional five-year CVD risk threshold of 10 percent. The main side effect in the model was an increased type 2 diabetes risk due to statin use. This trade-off was quantified in quality-adjusted life years (QALYs). Incremental cost-effectiveness ratios (ICERs) were compared to Australia’s AUD28,000 (EUR15,631) per QALY willingness-to-pay threshold, with financial outcomes adjusted annually at a three percent discount rate.

**Results:**

Early intervention was effective in preventing CVD across almost all risk profiles, resulting in increased QALYs and life expectancy for a significant portion of the population (37/54 females and 44/54 males in QALYs; 46/54 females and 47/54 males in life expectancy). Moreover, it was cost effective in various cases (5/54 females and 17/54 males). Notably, immediate intervention showed a favorable ICER in individuals who exhibit moderate long-term risk that may not be captured by short-term assessments.

**Conclusions:**

This study highlighted the urgency of reevaluating CVD prevention strategies within HTA, demonstrating that broader, more inclusive preventive interventions are not only effective but cost-effective as well. It signals the need for a significant shift to prioritize CVD, the top cause of global morbidity and mortality.

